# Melatonin Enhances Palladium-Nanoparticle-Induced Cytotoxicity and Apoptosis in Human Lung Epithelial Adenocarcinoma Cells A549 and H1229

**DOI:** 10.3390/antiox9040357

**Published:** 2020-04-24

**Authors:** Sangiliyandi Gurunathan, Muniyandi Jeyaraj, Min-Hee Kang, Jin-Hoi Kim

**Affiliations:** Department of Stem Cell and Regenerative Biotechnology, Konkuk University, Seoul 05029, Korea; muniyandij@yahoo.com (M.J.); pocachippo@gmail.com (M.-H.K.)

**Keywords:** palladium nanoparticles, melatonin, cytotoxicity, oxidative stress, mitochondrial dysfunctions, DNA damage, apoptosis

## Abstract

Palladium nanoparticles (PdNPs) are increasingly being used in medical and biological applications due to their unique physical and chemical properties. Recent evidence suggests that these nanoparticles can act as both a pro-oxidant and as an antioxidant. Melatonin (MLT), which also shows pro- and antioxidant properties, can enhance the efficacy of chemotherapeutic agents when combined with anticancer drugs. Nevertheless, studies regarding the molecular mechanisms underlying the anticancer effects of PdNPs and MLT in cancer cells are still lacking. Therefore, we aimed to investigate the potential toxicological and molecular mechanisms of PdNPs, MLT, and the combination of PdNPs with MLT in A549 lung epithelial adenocarcinoma cells. We evaluated cell viability, cell proliferation, cytotoxicity, oxidative stress, mitochondrial dysfunction, and apoptosis in cells treated with different concentrations of PdNPs and MLT. PdNPs and MLT induced cytotoxicity, which was confirmed by leakage of lactate dehydrogenase, increased intracellular protease, and reduced membrane integrity. Oxidative stress increased the levels of reactive oxygen species (ROS), malondialdehyde (MDA), nitric oxide (NO), protein carbonyl content (PCC), lipid hydroperoxide (LHP), and 8-isoprostane. Combining PdNPs with MLT elevated the levels of mitochondrial dysfunction by decreasing mitochondrial membrane potential (MMP), ATP content, mitochondrial number, and expression levels of the main regulators of mitochondrial biogenesis. Additionally, PdNPs and MLT induced apoptosis and oxidative DNA damage due to accumulation of 4-hydroxynonenal (HNE), 8-oxo-2′-deoxyguanosine (8-OhdG), and 8-hydroxyguanosine (8-OHG). Finally, PdNPs and MLT increased mitochondrially mediated stress and apoptosis, which was confirmed by the increased expression levels of apoptotic genes. To our knowledge, this is the first study demonstrating the effects of combining PdNPs and MLT in human lung cancer cells. These findings provide valuable insights into the molecular mechanisms involved in PdNP- and MLT-induced toxicity, and it may be that this combination therapy could be a potential effective therapeutic approach. This combination effect provides information to support the clinical evaluation of PdNPs and MLT as a suitable agents for lung cancer treatment, and the combined effect provides therapeutic value, as non-toxic concentrations of PdNPs and MLT are more effective, better tolerated, and show less adverse effects. Finally, this study suggests that MLT could be used as a supplement in nano-mediated combination therapies used to treat lung cancer.

## 1. Introduction

Melatonin is a pineal hormone that is primarily synthesized and secreted by the pineal gland from the essential precursor tryptophan. Melatonin is a multifaceted molecule that shows neuroprotective, anticancer, anti-inflammatory, and antioxidant activity [1]. MLT is responsible for synchronizing the circadian rhythm of physiological functions in mammals and plays a direct role in mitochondrial homeostasis [2]. MLT acts as an endogenous redox modulator, regulating antioxidant enzymes and exhibiting not only anticancer activity, but also protective effects against several diseases that lead to mitochondrial dysfunction [3]. MLT prevents apoptosis through a variety of well-recognized mechanisms; conversely, it shows anti-proliferative, anti-angiogenic, pro-apoptotic, and immunomodulatory properties in many cancer types [4,5]. The use of MLT has been growing very rapidly, as it enhances the success rate of chemotherapy with no side effects [6,7]. MLT also protects cells from adverse conditions caused by anticancer drugs. For example, MLT inhibited doxorubicin (DOX)-induced senescence of A549 human lung cancer cells and IMR90 normal lung cells in a dose-dependent manner, blocked the DOX-induced G2/M cell-cycle phase arrest, reduced the expression of cyclinB and cdc2, and reduced the levels of DOX-induced ROS, mitochondrial respiration, and loss of mitochondrial membrane potential [8]. Additionally, MLT has been shown to inhibit the migration of A549 human lung adenocarcinoma cells by reducing the expression level of osteopontin (OPN), myosin light-chain kinase (MLCK), and phosphorylation of MLC in A549 cells, while enhancing the expression of occludin [9].

In comparison to bulk materials, nanomaterials have unique advantageous properties, including optical, thermal, mechanical, and magnetic properties. Recently, diverse techniques have been developed to synthesize many metal nanoparticles (NPs). Among these techniques, biological methods seem to be preferred, as the resulting nanoparticles produced are soluble, biocompatible, sustainable, chemical-free, cost-effective, and eco-friendly. Such synthesized nanoparticles are used for multiple purposes, such as anticancer therapy, bio sensing, cell imaging, drug delivery, and clinical treatment. Palladium nanoparticles (PdNPs) play a significant role in nanomedicine and nanodiagnostics due to their efficient and broad catalytic activities, particularly in chemical reactions of therapeutic relevance [10]. Thus, PdNPs are frequently used in catalysis, dental materials, surgical instruments, biomedicine, ultrastructural immunolabeling, and as biosensors [11,12,13,14,15].

PdNPs and palladium-based materials such as dithiocarbamate Pd(II) complexes and quinolinic acid Pd(II) complexes exhibit high antitumor activity against cisplatin-resistant cancer cells [16,17]. PdNPs are able to catalyze the conversion of inactive prodrugs into toxic drugs in cells, thus enabling local chemotherapy [10]. At low concentrations, PdNPs induce autophagy, and at high concentrations, they induce autophagic flux blockade in Hela cells [18]. Moreover, PdNPs exhibits anticancer properties against several types of cancer cells, such as human ovarian cancer cells [19], human cervical cancer cells [20], and human breast cancer cells [21]. Recently, Gurunathan et al. (2019) [22] reported that in human ovarian cancer cells, PdNPs could potentially modulate genes involved in nucleosome assembly, telomere organization, and rDNA chromatin silencing.

Combination therapy is of great interest within the field of nanomedicine, particularly, the combination of MLT with anticancer drugs. For example, MLT promoted puromycin-induced apoptosis with activation of caspase-3 and 5′-adenosine monophosphate-activated kinase-alpha in human leukemia HL-60 cells [23]. The combination of MLT with cisplatin increased cytotoxicity, apoptosis, and cell-cycle arrest in a human lung adenocarcinoma cisplatin-sensitive cell line (SK-LU-1). In comparison to treatment with cisplatin alone, the combined treatment increased apoptosis by elevating mitochondrial membrane depolarization, activating caspases-3/7, and inducing cell cycle arrest in the S phase [24]. MLT suppresses lung cancer metastasis by inhibiting epithelial–mesenchymal transition, which is mediated by MT1 receptors and PLC, p38/ERK, and β-catenin signaling cascades [25]. Additionally, the anticancer effects of MLT act on different phases of carcinogenesis, including initiation, progression, and spread [26,27]. The combination of MLT with sorafenib synergistically inhibited proliferation and induced apoptosis by activating caspase-3 and the JNK/c-jun pathway of human hepatocarcinoma cells (HCC) [28]. MLT also has been reported to enhance the cytotoxic effects of rapamycin in head and neck squamous cell carcinoma (HNSCC) cells [29].

The combination of MLT with fluorouracil has been shown to stimulate cells and enhance the antiproliferative effect of these compounds by inhibiting survival pathways [30]. However, the potential effect of combining PdNPs and MLT in cancer therapy still remains unclear, and an in-depth exploration of the impact of PdNPs and MLT on adenocarcinoma is necessary. Several studies have reported the antioxidant property of MLT via its inhibition the proliferation, migration, and production of ROS [9,19,31,32], thus increasing caspase activity [33]. However, detailed studies related to combination of both PdNPs and MLT is remain elusive. Thus, we aimed to evaluate the effect of combining synthesized PdNPs with MLT, a natural molecule. We expected to determine the biological mechanisms related to its anticancer effects, including cell viability, proliferation, and morphology. We also aimed to determine its cytotoxicity, oxidative stress, mitochondrial dysfunctions, DNA damage, and apoptosis in human alveolar basal epithelial A549 cells. 

## 2. Materials and Methods

### 2.1. Materials

The human lung adenocarcinoma cell lines A549 and H1299 were obtained from the Korean Cell Bank (Seoul, Korea). Human epithelial lung cells (L132) were obtained from the Korean Cell Bank (Seoul, Korea). All cells were grown in 75 cm^2^ tissue culture flasks (Corning, New York, NY, USA) at 37 °C with 5% CO_2_ and 95% relative humidity. PdCl_2_ was purchased from Sigma-Aldrich (St. Louis, MO, USA). Penicillin–streptomycin, trypsin–EDTA, DMEM, fetal calf serum (FCS), and antibiotic–anti-mycotic reagents were obtained from Life Technologies/Gibco (Grand Island, NY, USA). The in vitro toxicology assay kit and the reagent kits used to measure oxidative stress markers, such as malondialdehyde, protein carbonyl contents, oxidants, and antioxidants assay kits, were purchased from Sigma-Aldrich (St. Louis, MO, USA). The lipid hydroperoxide assay kit (Catalog No.705003) and the 8-isoprostane kit (Catalog No.516351) were purchased from Cayman Chemical Company. Melatonin was dissolved in 0.5% of DMSO. All chemicals were purchased from Sigma-Aldrich unless otherwise stated. 

### 2.2. Synthesis and Characterization of PdNPs

PdNPs were prepared using resveratrol, according to Gurunathan et al. [19]. Resveratrol (1 mg) was suspended in 90 mL of sterile distilled water, mixed for 5 min, and then used in the synthesis of PdNPs. The resveratrol solution was combined with 10–100 mL of a 1 mM aqueous PdCl_2_ solution, and then incubated for 6 h at 60 °C with constant stirring. The reduction reaction occurred rapidly and changed the color of the solution from light brown to bright brown. Purification and characterization was carried out according to a method described previously [19]. 

### 2.3. Cell Culture and Exposure to MLT and PdNPs

Human lung epithelial adenocarcinoma cells were cultured in DMEM supplemented with 10% FBS and 100 U/mL penicillin–streptomycin at 5% CO_2_ and 37 °C. The medium was replaced three times a week, and the cells were passed at subconfluent levels. At 75% confluence, cells were harvested with 0.25% trypsin and were sub-cultured into either 75 cm^2^ flasks, 6 well plates, or 96 well plates, according to the requirements. Cells were allowed to attach to the surface for 24 h prior to treatment. A 100 μL aliquot of cells prepared at a density of 1 × 10^5^ cells/mL was plated in each of the 96 well plates. After 24 h, the culture medium was replaced with medium containing the IC_25_ concentrations of the all substances, as follows: 2.5 µM of PdNPs, 0.75 mM of MLT, 2.5 µM of PdNPs combined with 0.75 mM of MLT (PdNPs + MLT), or 5 µM of DOX. Dose-dependent effects were assessed using various concentration of PdNPs (1–10 µM), MLT (0.25–2.5 mM), and DOX (2.5–25 µM) for 24 h.

### 2.4. Cell Viability

Cell viability was measured using a Cell Counting Kit-8 (CCK-8; CK04-01, Dojindo Laboratories, Kumamoto, Japan). Briefly, A549 cells were plated in 96 well flat-bottomed culture plates containing the following substances for 24 h: 2.5 µM of PdNPs, 0.75 mM of MLT, and 2.5 µM of PdNPs combined with 0.75 mM of MLT or 5 µM of DOX. After 24 h at 37 °C and 5% CO_2_ in a humidified incubator, 10 μL of a CCK-8 solution was added to each well. The plates were then incubated for another 2 h at 37 °C. Absorbance was measured at 450 nm using a microplate reader (Multiskan FC; Thermo Fisher Scientific Inc., Waltham, MA, USA).

### 2.5. Cell Proliferation

Cells were incubated with 2.5 µM of PdNPs, 0.75 mM of MLT, 2.5 µM of PdNPs combined with 0.75 mM of MLT, or 5 µM of DOX for 24 h. The 5-bromo-2′-deoxyuridine (BrdU) labeling solution was added to the culture medium 2 h before the incubation period ended. Cells were fixed and the level of incorporated BrdU was determined using the Cell Proliferation ELISA BrdU assay kit (Roche), following the manufacturer’s instructions. The proliferation activity of untreated cells at 0 h was considered to be 100%. 

### 2.6. Membrane Integrity

We evaluated the membrane integrity of A549 cells using an LDH Cytotoxicity Detection Kit. Briefly, cells were exposed to 2.5 µM of PdNPs, 0.75 mM of MLT, 2.5 µM of PdNPs combined with 0.75 mM of MLT, or 5 µM of DOX for 24 h. Subsequently, 100 μL of cell-free supernatant from each well was transferred into the wells of a 96 well plate and 100 μL of the LDH reaction mixture was added to each well. After 3 h of incubation under standard conditions, the optical density of the final solution was determined at a wavelength of 490 nm using a microplate reader. These procedures were carried out three times.

### 2.7. Cell Mortality

Cell mortality was evaluated using the trypan blue assay according to Gurunathan et al. [34]. A549 cells were plated into wells of 6 well plates (1 × 10^5^ cells per well) and incubated for 24 h with 2.5 µM of PdNPs, 0.75 mM of MLT, 2.5 µM of PdNPs combined with 0.75 mM of MLT, or 5 µM of DOX. Cells cultured in medium without MLT, PdNPs, or DOX were used as controls. After 24 h, cells were detached with 300 µL trypsin–EDTA solution and both adherent and suspended cells were collected. The mixture of the supernatant and detached cells was centrifuged at 1200 rpm for 5 min. The pellet was mixed with 700 µL of trypan blue solution and dispersed. After 5 min of staining, the cells were counted using a cytometer; viable cells were unstained and dead cells were stained blue. Three independent experiments were performed of which we calculated the mean and standard deviation. Cell mortality is expressed as the percentage of dead cells in relation to the respective control.

### 2.8. Assessment of Dead-Cell Protease Activity

A dead-cell protease activity assay was performed according to a method described previously [35]. The cytotoxicity was evaluated by treating the cells with 2.5 µM of PdNPs, 0.75 mM of MLT, 2.5 µM of PdNPs combined with 0.75 mM of MLT, or 5 µM of DOX for 24 h. The cytotoxicity was determined by association of intracellular proteases with a luminogenic peptide substrate (alanyl–alanylphenylalanyl–aminoluciferin). The degree of protease reaction can measure dead-cell protease activity. As a control, we treated cells with 1% Triton X-100 to exclude the background value of the medium color. Luminogenic peptide substrate (5 μL) was added to each well, and luminescence was measured to determine the number of dead cells. The peptide substrate was incubated for 15 min at 37 °C. The luminescence was measured with a luminescence counter (Perkin Elmer, Waltham, MA, USA). 

### 2.9. Determining ROS, MDA, NO, Carbonylated Protein Levels, Lipid Hydroperoxide, and 8-Isoprostane

ROS were estimated according to Gurunathan et al. [36]. A549 cells were seeded into wells of 24 well plates at a density of 5 × 10^4^ cells per well and cultured for 24 h. After washing twice with phosphate-buffered saline (PBS), fresh medium containing 2.5 µM of PdNPs, 0.75 mM of MLT, 2.5 µM of PdNPs combined with 0.75 mM of MLT, or 5 µM of DOX was added and cells were incubated for 24 h. Cells were supplemented with 20 μM DCFH2-DA, and incubation continued for 30 min at 37 °C. Cells were then rinsed with PBS (2 mL of PBS was added to each well) and the fluorescence intensity was determined using a Gemini EM spectrofluorometer (Molecular Devices, Sunnyvale, CA, USA) at an excitation wavelength of 485 nm and an emission wavelength of 530 nm. MDA levels were determined using a thiobarbituric-acid-reactive substance assay based on Gurunathan et al. [36]. NO production was quantified spectrophotometrically using the Griess reagent (Sigma-Aldrich). Absorbance was measured at 540 nm and the nitrite concentration was determined using a calibration curve prepared with sodium nitrite according to Chen et al. [37]. Additionally, carbonylated protein content was measured based on Maisonneuve et al. [38] and the lipid hydroperoxide and 8-isoprostane were measured according to de Sousa-Leal et al. [39]. 

### 2.10. Mitochondrial Membrane Potential (MMP)

We measured the mitochondrial membrane potential (MMP) using the JC-1 cationic fluorescent indicator according to the manufacturer’s instructions (Molecular Probes, Eugene, OR, USA). A549 cells were incubated with 2.5 µM of PdNPs, 0.75 mM of MLT, 2.5 µM of PdNPs combined with 0.75 mM of MLT, or 5 µM of DOX for 24 h followed by 10 μM JC-1 at 37 °C for 15 min. Cells were then washed and resuspended in PBS, after which fluorescence intensity was measured. Mitochondrial membrane potential was expressed as the ratio of the fluorescence intensity of JC-1 aggregates to that of the monomers. 

### 2.11. ATP

A549 cells were incubated with the above mentioned IC25 concentrations for 24 h, after which ATP was measured according to the manufacturer’s instructions (Catalog No., MAK135; Sigma-Aldrich, St. Louis, MO, USA). Decreased levels of ATP indicated increased cytotoxicity. 

### 2.12. Mitochondrial DNA Copy Number

We determined mitochondrial dysfunction of A549 cells exposed to 2.5 µM of PdNPs, 0.75 mM of MLT, 2.5 µM of PdNPs combined with 0.75 mM of MLT, or 5 µM of DOX for 24 h using real-time PCR amplification and assessment of mitochondrial copy numbers. The following primers were used to determine copy numbers: mtDNA forward—CCTATCACCCTTGCCATCAT and mtDNA reverse—AGGCTGTTGCTTGTGTGAC. Nuclear DNA was quantified using the primer set that detects the *Pecam* gene on chromosome 6: forward—ATGGAAAGCCTGCCATCATG and reverse—TCCTTGTTGTTCAGCATCAC [40].

### 2.13. Enzyme-Linked Immunosorbent Assay

4-hydroxynonenal (HNE), 8-oxo-2′-deoxyguanosine (8-OhdG), and 8-hydroxyguanosine (8-OHG) were measured according to the literature [41,42] and to the manufacturer’s instructions (Trevigen, Gaithersburg, MD, USA). ELISA kits were used to measure concentrations of 4-hydroxynonenal and of 8-hydroxy-2′-deoxyguanosine (8-OHdG and 8-OHG). HNE, 8-OHdG, and 8-OHG were measured in A549 cells exposed to 2.5 µM of PdNPs, 0.75 mM of MLT, 2.5 µM of PdNPs combined with 0.75 mM of MLT, or 5 µM of DOX for 24 h.

### 2.14. Reverse Transcription-Quantitative Polymerase Chain Reaction (RT-qPCR)

Total RNA was extracted from LNCaP cells treated with 2.5 µM of PdNPs, 0.75 mM of MLT, 2.5 µM of PdNPs combined with 0.75 mM of MLT, or 5 µM of DOX for 24 h using the PicoPure RNA isolation kit (Arcturus Bioscience, Mountain View, CA, USA). Samples were prepared according to the manufacturer’s instructions. Real-time RT-qPCR was conducted using a Vill7 device (Applied Biosystems, Foster City, CA, USA) and SYBR Green as the double-stranded DNA-specific fluorescent dye (Applied Biosystems). Target gene expression levels were normalized to the expression of glyceraldehyde-3-phosphate dehydrogenase (GAPDH), which was unaffected by treatments. The real-time qRT-PCR primer sets are shown in Table S1.

### 2.15. Cell Apoptosis

To detect apoptotic cells, we used A549 cells treated with 2.5 µM of PdNPs, 0.75 mM of MLT, 2.5 µM of PdNPs combined with 0.75 mM of MLT, or 5 µM of DOX for 24 h. Approximately 1 μL of a dye mixture containing acridine orange (AO) and ethidium bromide (EtBr) was mixed with 9 mL of a cell suspension (1 × 10^5^ cells per ml) on a clean microscope coverslip. The cells were extracted, washed with phosphate-buffered saline (PBS; pH 7.2), and stained with 1 mL of AO/EtBr. Cells were then incubated for two min, washed twice with PBS (5 min each), and observed under a fluorescence microscope at 400 × magnification with an excitation filter at 480 nm. 

### 2.16. Measurement of Caspase 9/3 Activity

The caspase-3 activity was measured according to the method described previously [43]. A549 and H1229 cells were treated with 2.5 µM of PdNPs, 0.75 mM of MLT, 2.5 µM of PdNPs combined with 0.75 mM of MLT, or 5 µM of DOX for 24 h, and then the activity of caspase-3/9 was measured in the cancer cells using a kit from Sigma-Aldrich Co., according to the manufacturer’s instructions. The calorimetric assay was based on the hydrolysis of the caspase-9/3 substrate by caspase-9/3, resulting in the release of the p-nitroaniline (pNA) moiety. The concentration of pNA released from the substrate was calculated from the absorbance values at 405 nm. 

### 2.17. Statistical Analysis

All experiments were repeated at least three times, and data are shown as mean ± SD. Data were analyzed by *t*-tests, multivariate analysis, or one-way analysis of variance (ANOVA) followed by Tukey’s test for multiple comparisons to determine the differences between groups.

## 3. Results and Discussion

### 3.1. Synthesis and Characterization of PdNPs

**Figure 1:**
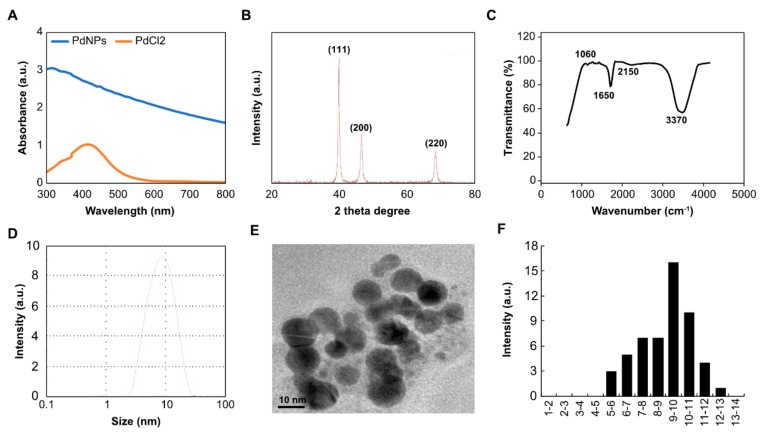
Synthesis and characterization of palladium nanoparticles (PdNPs) using resveratrol. (**A**) Ultraviolet-visible spectroscopy (UV-vis) absorption spectra of resveratrol-mediated synthesis of PdNPs (**B**). X-ray diffraction patterns of PdNPs (**C**). Fourier-transform infrared spectroscopy (FTIR) spectra of PdNPs (**D**). Size distribution analysis of PdNPs using dynamic light scattering (DLS) (**E**). Transmission electron microscopy (TEM) images of PdNPs (**F**). Histograms showing particle size distribution. All experiments were performed three times to obtain reproducible results. The data represent the results of a representative experiment.

PdNPs were synthesized using RES solution combined with 1 mM of aqueous PdCl_2_ solution with resveratrol (1 mg), and then incubated for 6 h at 60 °C with constant stirring. The reduction reaction occurred rapidly and was indicated by a solution color change from light to bright brown. The absorption spectrum of PdCl_2_ was around 420 nm due to the absorption of Pd(II) ions in PdCl_2_, which were reduced to Pd(0) NPs. The absorption band at 320 nm indicated that PdNPs were formed and Pd(II) ions were reduced to Pd(0) NPs (Figure 1A). 

The crystallinity of the synthesized PdNPs was examined by X-ray diffraction (XRD) analysis. Three distinct reflections were present in the diffraction at 40.20 (111), 46.60 (200), and 68.70 (220) (Figure 1B), which represented the face centered cubic (fcc) structure of PdNPs [19]. The strong reflection at (111) may specify the desired development track of nanocrystals [44,45]. Based on the half width of the (111) reflection, the average crystallite size (~10 nm) of PdNPs was calculated using the Debye–Scherer equation.

Fourier-transform infrared spectroscopy (FTIR) was performed to determine the role of RES as a stabilizing and reducing agent during the synthesis of PdNPs. Examining the FTIR spectra of RES, we observed that PdNPs showed an absorption peak at ~3370 cm^−1^ (Figure 1C), indicating the presence of hydroxyl groups and thus the existence of several oxygen-including functional groups, such as carboxylic, epoxy, carbonyl, and hydroxyl groups. The peak at ~2150 cm^−1^ indicated an asymmetrical stretching of C=O, the peak at ~1650 cm^−1^ indicated the stretching of C=C, and the peak at ~1060 cm^−1^ indicated the C–O stretching. RES has been previously used as both a reducing and a stabilizing agent to synthesize reduced graphene oxide, which is a rich source of phenolic compounds and glycosides. The presence of secondary metabolites, such as phenolic compounds and their glycosides, in RES could be due to the reduction of Pd(II) ions. Similarly, Shaik et al. [45] reported in 2017 that extracts of *O. vulgare* L. containing phenolic compounds were responsible for the redox-type reaction that takes place as Pd(II) is reduced to Pd(0) NPs. Therefore, the formation of PdNPs is due to polyphenols of RES, which are essential in the reduction of Pd(II) ions and also act as a stabilizing agent for the synthesized PdNPs.

Finally, we determined the size and morphology of synthesized PdNPs using dynamic light scattering and TEM, respectively. Size ranged between 2 and 20 nm with an average size of 10 nm (Figure 1D). An expanded image revealed that all the particles formed were significantly spherical in shape with an average size of 10 nm (Figure 1E). The diameter of PdNPs varied from nearly 2 to 13 nm with an average diameter of 10 nm (Figure 1F). The size distribution and TEM analysis revealed that particle sizes were consistent. 

### 3.2. Effects of PdNPs, MLT, and DOX on A549 Cell Viability and Proliferation

**Figure 2:**
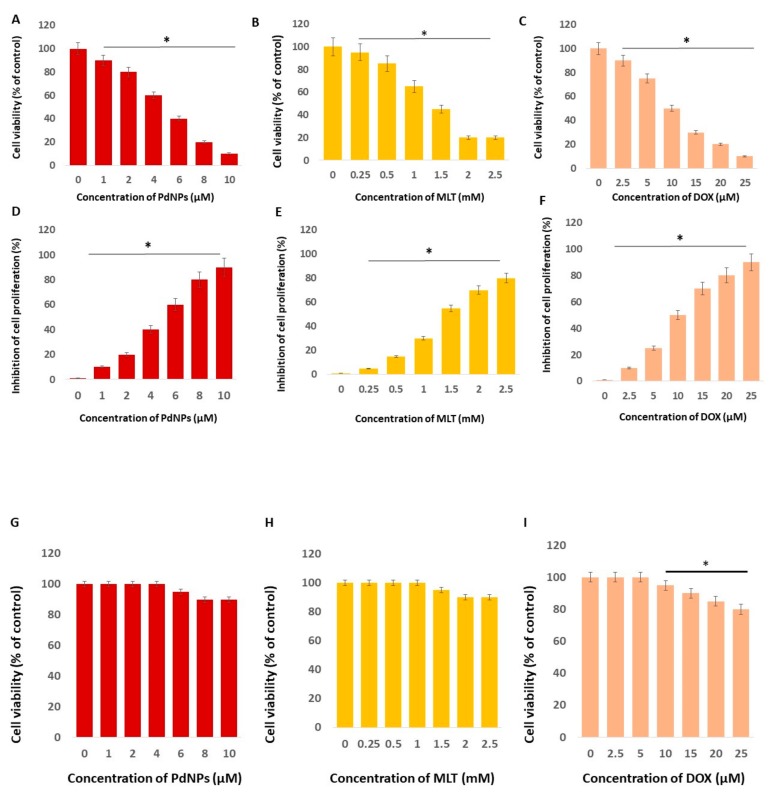
Effects of PdNPs, MLT, and DOX on cell viability and proliferation. Cell viability (**A–C**) and cell proliferation (**D–F**) of human adenocarcinoma cells (A549) exposed to different concentrations of PdNPs, MLT, or DOX for 24 h. Cells were treated with PdNPs at concentrations ranging from 1 to 10 µM, MLT at concentrations ranging from 0.25 to 2.5 mM, or DOX at concentrations ranging from 2.5 to 25 µM. Results are shown as the mean ± standard deviation of three independent experiments. The treated groups showed statistically significant differences from the control group per Student’s *t*-test. * *p* < 0.05 was considered significant. Cell viability (**G–I**) of human normal lung cells (L132) exposed to different concentrations of PdNPs, MLT, or DOX for 24 h. Cells were treated with PdNPs at concentrations ranging from 1 to 10 µM, MLT at concentrations ranging from 0.25 to 2.5 mM, or DOX at concentrations ranging from 2.5 to 25 µM. The treated groups showed no significant differences from the control group per Student’s *t*-test.

In order to determine the IC_25_ concentration, we first determined the effect of different concentrations of PdNPs (1–10 µM), MLT (0.25–2.5 mM), and DOX (2.5–25 µM) in A549 cells. PdNPs, MLT, and DOX all showed a concentration-dependent effect in A549 cells (Figure 2). The cell viability of A549 cells decreased significantly when exposed to PdNPs, at concentrations ranging from 1 to 10 µM (Figure 2A), and to MLT at concentrations ranging from 0.25 to 2.5 mM, with a more pronounced effect at concentrations between 1 and 2 mM (Figure 2B). Cell viability also declined when cells were exposed to DOX (Figure 2C), which was used as a positive control. Moreover, A549 cells exposed to PdNPs showed a significant reduction in cell proliferation, with up to 90% reduction at concentrations of 10 µM (Figure 2D). A similar trend was also observed in cells treated with MLT (Figure 2E) and DOX (Figure 2F). It has been recently reported that the cytotoxic activity of MLT increases in a concentration-dependent manner; the cell viability of A431 and CCD-1079Sk cells exposed to 0.062 mM of MLT was 130% greater in relation to its control, whereas at a concentration of 0.125 mM, cell viability was not affected. Thus, higher doses of melatonin (MLT) resulted in greater mortality of cancer cells in relation to non-cancer cells. In A549 cells, the half maximal inhibitory concentration (IC_50_) of PdNPs was 5 µM, 1.5 mM for MLT, and 10 µM for DOX. Therefore, we selected IC_25_ concentrations of PdNPs (2.5 µM), MLT (0.75 mM), PdNPs combined with MLT (2.5 + 0.75 mM), and DOX (5.0 µM) for further study. A previous study reported the bioavailability of melatonin in both male and female healthy volunteers. The maximum plasma MLT concentration C_max_ ± S.D. was found at 243.7 ± 124.6 pg/mL and 623.6 ± 575.1 pg/mL for male and female subjects, respectively, while the mean values for the area under the plasma concentration–time curve (AUC) were 236 ± 107 pg.h/mL and 701 ± 645 pg.h/mL [46]. 

In order to avoid harmful effects of both PdNPs and MLT to normal cells, the study was performed to analyze dose-dependent effect of PdNPs, MLT, and DOX on normal lung cells L132. Concentration responses were assessed to compare the effects of PdNPs, MLT, and DOX (which was used as positive control) between normal and cancer cells. L132 normal lung cells were incubated with various concentrations of PdNPs (1–10 µM), MLT (0.25–2.5 mM), or DOX (2.5–25 µM) in L132 cells for 24 h. Following incubation, the effects of PdNPs, MLT, and DOX were determined by the CCK-8 cell viability assay. Cell viability in all cell cultures were greater than 95% before all experiments. After the addition of either PdNPs or MLT or DOX, the cell viability was the same as the control in the tested concentration. Interestingly, no significant toxicity was observed in the cells treated with either PdNPs, MLT, or DOX (Figure 2G–I). Although PdNPs, MLT, and DOX decreased the cell viability at higher concentrations, the effect was not significant compared to the control group. For this reason, as shown in Figure 2G–I, the non-toxic concentration for normal cells and mildly toxic concentration to cancer cells were selected for further study. 

Pd–Au heterostructures irradiated with an 808 nm laser light destroyed HeLa cancer cells [47]. Biologically synthesized PdNPs potentially induced cytotoxicity in human ovarian cancer cells by reducing cell viability and cell proliferation and increasing ROS [19]. Dendrimer-encapsulated PdNPs (DEPdNPs) nanoparticles possessed a sub-5 nm size and exhibited an excellent photothermal effect [48]. The combination of PdNPs and trichostatin-A showed significant cytotoxicity in human cervical cancer cells and also affected the oxidative stress, mitochondrial membrane potential (MMP), caspase-3/9 activity, and expression of pro- and anti-apoptotic genes [20]. Additionally, flower extracts of *Moringa oleifera* assisted PdNPs in reducing cell viability and proliferation of human lung carcinoma cells (A549) and peripheral lymphocytes non-cancer cells [49]. PdNPs inhibited cell growth in fibroblasts and lung epithelial cells; however, the inhibition of cell growth was not associated with the induction of apoptosis [50]. Extracts of *Pelargonium graveolens* exceeded PdNPs in reducing the cell viability and proliferation of human leukemia cell lines (K562). Recently, Gurunathan et al. [22] reported that PdNPs induced cytotoxicity and PdNPs-exposed SKOV3 cells showed several dysregulated pathways, particularly those involved in nucleosome assembly, telomere organization, and rDNA chromatin silencing.

### 3.3. PdNPs + MLT Decreased Cell Viability and Proliferation

**Figure 3:**
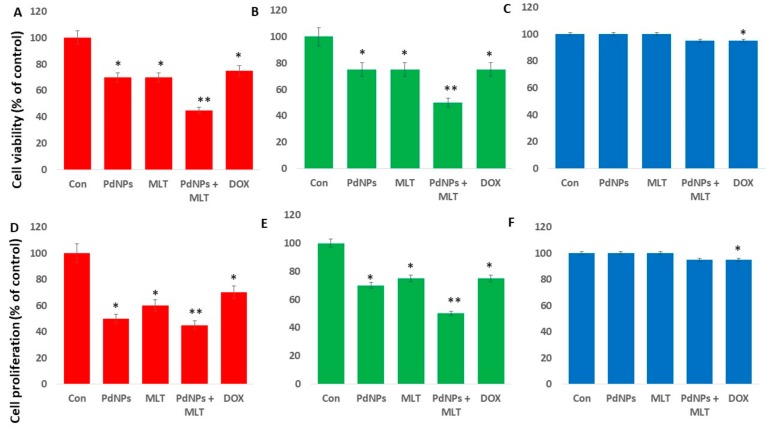
Effects PdNPs + MLT on cell viability and proliferation. Cell viability of A549 (**A**) H1229 (**B**) and L132 (**C**) cells and cell proliferation of human adenocarcinoma cells A549 (**D**), H1229 (**E**), and L132 (**F**) in each of the following treatments: control, 2.5 µM PdNPs, 0.75 mMMLT, 2.5 µM PdNPs combined with 0.75 mM MLT, and 5 µM DOX. Results are shown as the mean ± standard deviation of three independent experiments. The treated groups showed statistically significant differences from the control group per Student’s *t*-test. * *p* < 0.05 was considered significant; ** *p* < 0.01 was considered highly significant.

There are no reports on the effects of the combined use of PdNPs and MLT on A549 cells. In order to address the effect of PdNPs combined with MLT on human lung cancer cells, we selected two different type of human lung cancer cells, A549 and H1229, and also a normal lung cell line called L132. All these cells were exposed to PdNPs (2.5 µM), MLT (0.75 mM), PdNPs combined with MLT (2.5 + 0.75 mM), or DOX (5.0 µM) for 24 h. All treatments significantly affected cell viability of both A549 and H1229 cells (Figure 3A,B), where cell viability reduced by 25% when cells were treated with PdNPs, MLT, or DOX separately, and by 60% when cells were treated with the combination of PdNPs and MLT. Our findings suggest that PdNPs (at concentrations ≥ 1 µM) and MLT (at concentrations ≥ 0.5 µM) reduced the proliferation of human lung cancer cells. The normal lung cells treated with PdNPs (2.5 µM), MLT (0.75 mM), PdNPs combined with MLT (2.5 + 0.75 mM), or DOX (5.0 µM) for 24 h showed no significant toxicity (Figure 1C). Similarly, the combination effect of PdNPs and MLT on cell proliferation of A549, H1229, and L132 cells were assessed. The results showed that the cell proliferation was significantly compromised, which was corroborated with cell viability assay (Figure 3D,E). Interestingly, there was no effect was observed in L132 cells (Figure 3F). Collectively, all these results indicate that either PdNPs, MLT, or a combination of both targets cancer cells rather than normal cells.

PdNPs have recently been reported to induce cytotoxicity against different types of cancer cells by affecting cell viability and proliferation [19,22]. PdNPs potentially induce loss of cell viability in a variety of cancer cells, including human ovarian cancer cells, peripheral lymphocytes, cervical cancer cells, fibroblasts, and human leukemia cell lines [19,22,49,50]. PdNPs combined with tubastatin-A have been reported to inhibit cell viability and proliferation in both human ovarian cancer cells and breast cancer cells [20,21]. Similarly, at higher concentrations, MLT reduces cell viability and proliferation in human lung cancer cells, but at lower concentrations, exhibits a pro-proliferative effect. The cytotoxic effect of melatonin at high doses was greater in cancer cells than in normal cells [51]. The combination of MLT with retinoic acid and somatostatin increased its effect on MCF-7 cell viability and proliferation [52], while the combination of MLT and valproic acid (VPA) inhibited proliferation of UC3 bladder cancer cells [53]. However, since we reduced undesired cytotoxic effects by using the IC_25_ concentration, we were not able to determine the precise combination of PdNPs and MLT needed to achieve the best results in human adenocarcinoma cells. 

### 3.4. MLT Enhanced the Cytotoxic Effect of PdNPs

**Figure 4:**
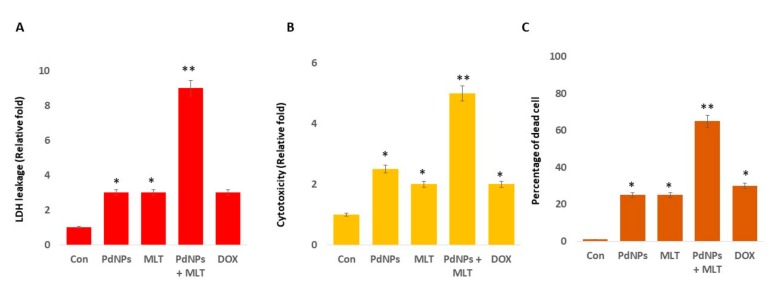
Effects of PdNPs + MLT in A549 cells. (**A**) LDH leakage, (**B**) cytotoxicity, and (**C**) percentage of dead cells in each of the following treatments: control, 2.5 µM PdNPs, 0.75 mM MLT, 2.5 µM PdNPs combined with 0.75 mM MLT, and 5 µM DOX. Results are shown as the mean ± standard deviation of three independent experiments. The treated groups showed statistically significant differences from the control group per Student’s *t*-test. * *p* < 0.05 was considered significant; ** *p* < 0.01 was considered highly significant.

We investigated the relationship between the anti-proliferative and cytotoxic effects of PdNPs and MLT in A549 cells. The cells were exposed to PdNPs (2.5 µM), MLT (0.75 mM), PdNPs and MLT (2.5 + 0.75 mM), or DOX (5.0 µM) for 24 h, and cytotoxicity markers such as LDH, intracellular protease, and cell mortality were quantified. In comparison to the control treatment, the treatment combining PdNPs with MLT significantly increased LDH leakage, indicating that this treatment enhanced cytotoxicity. Additionally, in comparison to all other treatments, the combination treatment (PdNPs + MLT) enhanced cell death, which might have been due to the disruption of cell membranes leading to the release of lactate dehydrogenase (LDH) (Figure 4A). Harms et al. [54] reported that a single-dose treatment of melatonin (dose ranging from 0.01 to 0.5 mM) in primary neuronal cultures induced LDH release, particularly at high concentrations, which could lead to complete loss of metabolic activity. In contrast, Liu et al. [53] observed that combining MLT with VPA significantly reduced LDH leakage, even though toxicity increased.

We also assessed the relationship between intracellular proteases and cell death. Our results revealed that the treatment combining PdNPs and MLT increased the amount of dead cell proteases (Figure 4B), in agreement with results focusing on cell viability and LDH leakage. To further confirm the cytotoxic effect of PdNPs + MLT, we measured the mortality rate of cells and observed that PdNPs + MLT could cause severe damage to the integrity of membranes, possibly leading to cell death (Figure 4C).

Several studies have reported that metal nanoparticles such as silver, gold, palladium, platinum, and zinc induce leakage of LDH and cell death in different cancer and non-cancer cells [19]. The combination of trichostatin-A + PdNPs showed a significant effect on cytotoxicity, oxidative stress, mitochondrial membrane potential (MMP), caspase-3/9 activity, and expression of pro- and anti-apoptotic genes in human cervical cancer cells [20], while the combination of tubastatin-A (TUB-A) + PdNPs had a greater effect on cell viability in relation to treatments where cells were treated with TUB-A or PdNPs alone. The combined treatment showed a greater effect on HDAC inhibition and enhanced apoptosis by regulating several cellular and biochemical changes in human breast cancer cells [21]. Combining MLT with all-trans retinoic acid and somatostatin further enhanced the effects of MLT on MCF-7 cell viability and growth, probably due to alterations in the Ca^2+^- and voltage-activated K+ (BK) channels and to substantial impairments of Notch-1 and epidermal growth factor (EGF)-mediated signaling [52]. Additionally, UC3 bladder cancer cells treated with MLT + VPA enhanced cytotoxicity by regulating cell death pathways [53]. Altogether, PdNPs + MLT induced leakage of LDH, intracellular protease, and cell death. 

### 3.5. Combining PdNPs with MLT Altered Cell Morphology

**Figure 5:**
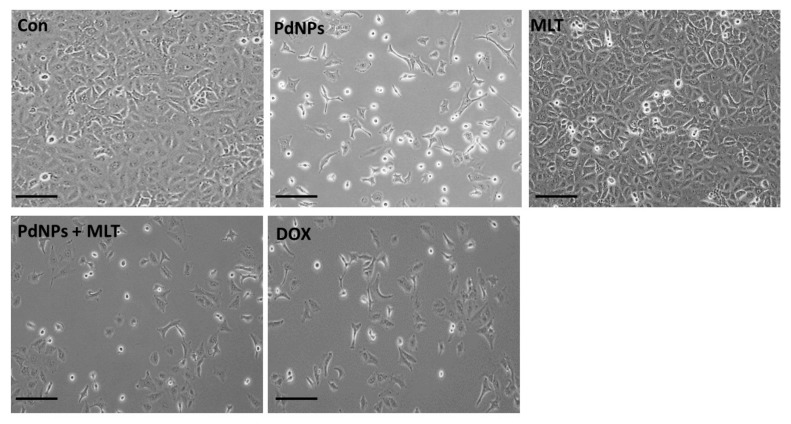
Combining PdNPs with MLT altered cell morphology in A549 cells. A549 cells were incubated with PdNPs (2.5 µM), MLT (0.75 mM), PdNPs and MLT (2.5 + 0.75 mM), or DOX (5.0 µM) for 24 h. Cell morphology was determined microscopically. Scale bar: 100 µm.

In order to analyze the influence of PdNPs and MLT on A549 cell morphology and to corroborate the cytotoxicity, the cells were cultured in the presence of PdNPs and MLT (2.5 µM + 0.75 mM), PdNPs (2.5 µM), MLT (0.75 mM), or DOX (5 µM) for 24 h. Alteration of cell morphology indicates apoptosis. We observed cell detachment, a greater number of unhealthy cells and a reduced number of cells in all treated cells (Figure 5), indicating that cell detachment was correlated to cell toxicity. The combination treatment (PdNPs + MLT) had a greater cytotoxic effect in comparison to the other treatments. It has been reported that PdNPs affect cell morphology in human ovarian cancer cells [19].

Cells exposed to PdNPs and MLT appeared to be fragmented and clumped together, exhibiting severe morphological alterations that could eventually lead to cell death. Furthermore, cells were observed to have a smaller size and a spherical shape, forming clusters that eventually detached from the surface. Similarly, Yuan et al. [21] observed significant changes in cell morphology when cells were treated with TUB-A and PdNPs. Furthermore, MLT has also been shown to alter cell morphology; Reddins et al. [55] reported that MLT altered the ultrastructure of mouse Leydig cells, possibly affecting their capacity to secrete steroids, while Ikeno et al. [56] reported that MLT induced structural changes of hippocampal neurons, increasing apical dendritic length and complexity and reducing the dendritic spine density. The combination of MLT with all-trans retinoic acid and somatostatin also caused a series of morphological alterations, including pyknotic nuclei, swollen mitochondria with disrupted cristae, autophagic vesicles, and dissolution of the plasma membrane associated with spillage of the cytoplasmic content. Tamoxifen 1 μM displayed features of apoptosis, mainly in cytoplasmic condensation, DNA fragmentation, and membrane blebbing [52]. Our results are consistent with other studies [19,21] and confirm that cell death is characterized by reduced and shrunken cells. Our findings suggest that PdNPs and MLT alter cellular morphology, which indicates their cytotoxicity. Morphological alterations such as distinct cell shrinkage and membrane blebbing strongly correlate with apoptosis.

### 3.6. Effect of PdNPs + MLT on Oxidative Stress Markers

**Figure 6:**
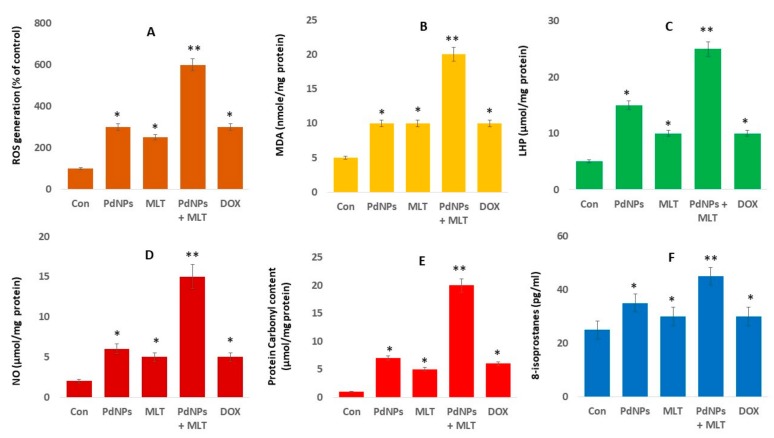
PdNPs + MLT increased production of oxidative stress markers. A549 cells were incubated with PdNPs (2.5 µM), MLT (0.75 mM), PdNPs and MLT (2.5 + 0.75 mM), or DOX (5.0 µM) for 24 h. (**A**) Treated A549 cells were analyzed by fluorescence microscopy. Spectrophotometric analysis of ROS using 2′,7′-dichlorodihydrofluorescein diacetate (DCFH-DA). (**B**). Malondialdehyde (MDA) concentration was measured using a thiobarbituric-acid-reactive substances assay and was expressed as nanomoles per gram of protein. (**C**) The lipid hydroperoxide ( LHPs) were extracted and quantified as indicated using the Lipid Hydroperoxide Assay Kit (Catalog No. 705003; Cayman Chemical Company). (**D**) Nitric oxide (NO) production was quantified spectrophotometrically using the Griess reagent and expressed as micromoles per gram of protein. (**E**) Protein carbonylation content was determined and expressed as micromoles per gram of protein. (**F**) 8-Isoprostane was quantified as described in the EIA Kit (Catalog No.516351, Cayman Chemical Company). Results are shown as the mean ± standard deviation of three independent experiments. Student’s *t-*test revealed a significant difference between treated and control cells (* *p* < 0.05). * *p* < 0.05 was considered significant; ** *p* < 0.01 was considered highly significant.

Metal nanoparticles such as silver, gold, platinum, palladium, and zinc oxide are toxic and cause cell death due to an excessive production of ROS. MLT has also been reported to increase ROS levels [57]. We determined the involvement of ROS in cells treated with PdNPs and MLT. Our results revealed that the combination treatment (PdNPs + MLT) increased levels of ROS compared both to untreated cells and to cells treated with PdNPs and MLT separately (Figure 6A). Previous studies have suggested that PdNPs induce oxidative stress in A375 malignant human skin melanoma cells [58] and in A2780 human ovarian cancer cells [19]. The excessive production of ROS induces DNA damage and apoptosis-mediated cell death, and could potentially lead to autophagy-mediated cell death [19]. 

One of the biological markers most frequently used to evaluate oxidative stress induced by nanoparticles is the level of lipid peroxidation. Given that malondialdehyde (MDA) is as a marker of lipid peroxidation, we quantified the level of MDA in cells of each treatment (Figure 6B). MDA was significantly greater in cells treated with PdNPs + MLT compared to untreated cells and to cells treated with PdNPs and MLT separately (Figure 6B), suggesting that the damage to the membrane was directly related to the amount of MDA. These findings indicate that ROS generation is clearly associated with increased levels of MDA [59]. Similarly, previous studies have reported that PdNPs increased levels of MDA in ovarian cancer cells [19], in malignant melanoma cells [58], in cervical cancer cells [20], and in breast cancer cells [21], which could lead to cell death given that MDA determines the level of plasma membrane oxidation—the initial processes in oxidative damage. Treating A549 cells with PdNPs + MLT significantly increased MDA levels, indicating that the combination of PdNPs with MLT may induce oxidative damage in cells. For example, cortical neurons treated with various concentrations of MLT (0.5, 1.0, 1.5, 2.0 mM) exhibited dose-dependent increases in the levels of MDA. Similarly, treatment with 300 nM staurosporine also increased MDA levels in cortical neuronal cells, as well as causing cell death. Interestingly, treatment with a low concentration of MLT (0.1 or 0.5 mM) did not prevent staurosporine-induced neuronal cell death [54]. Altogether, combined use of PdNPs and MLT potentially induces MDA via oxidative stress and ultimately leads to cell death.

The oxidative catabolism of polyunsaturated fatty acids, known as lipid hydroperoxidation (LHP), is a widely accepted mechanism of cellular injury and death [60]. The production of LHP and free radicals are deleterious processes related to toxicity. The most widely used method to evaluate oxidative catabolism of lipid membranes is to quantify lipid hydroperoxide (LHP), a stable aldehydic product of lipid peroxidation [60]. To further prove that the combination of PdNPs and MLT affected oxidative catabolism of polyunsaturated fatty acids, we measured the level of LHP. A549 cells were exposed to PdNPs (2.5 µM), MLT (0.75 mM), or PdNPs and MLT (2.5 + 0.75 mM) for 24 h, and a significant increase in the LHP levels was observed in all treated groups (Figure 6C). Our findings demonstrated that PdNPs + MLT can potentially increase the level of LHP, similarly to previous reports implicating LHP in the toxicity of metal nanoparticles such as AgNPs.

Nitric oxide (NO) plays a critical role in cell proliferation, cell differentiation, and cell death. We therefore quantified the effect of PdNPs and MLT on NO production in A549 cells and found that cells treated with PdNPs + MLT showed a significant increase in the production of NO compared to untreated cells (Figure 6D). Previous studies have reported that NO may activate biological signaling that cause cells apoptosis [61]. Platinum nanoparticles have been reported to increase the NO levels that ultimately cause cell death in human bone OS epithelial cells (U2OS) cells. NO has protective and neurotoxic effects in focal cerebral ischemia [62]. MLT increased the chemotherapeutic effect of 5-fluorouracil in colon cancer by suppressing PI3K/AKT and NF-κB/iNOS signaling pathways [63].

Analysis of protein carbonylation is a versatile and sensitive method to describe NP-induced oxidative stress. Therefore, we measured the level of protein carbonylation in the A549 cells exposed to PdNPs (2.5 µM), MLT (0.75 mM), or PdNPs + MLT (2.5 + 0.75 mM) for 24 h; all treated cells showed a significant increase in protein carbonylation (Figure 6E). AgNPs have been reported to induce protein carbonylation in THP-1 macrophages, primary neuronal cells [64], and a human colon epithelial cell line [65]. Gurunathan et al. [66] reported that platinum nanoparticles, doxorubicin, cisplatin, platinum nanoparticles, and doxorubicin induce protein carbonylation in human bone OS epithelial cells (U2OS). The amount of protein carbonylation indicates the severity of oxidative stress. Protein carbonylation in THP-1 macrophages exerts a strong cytotoxic effect and induces oxidative stress in NM-401. 

Oxidative stress seems to be one of the main mechanisms of cytotoxicity leading to cell death. To clarify the role of oxidative stress induced by PdNPs and MLT in A549 cells, we measured the oxidative stress markers as mentioned above. However, to evaluate the role of free radicals throughout the build-up of toxicity, more reliable markers were needed. 8-Isoprostane has been proposed as a reliable biomarker of oxidative stress, as it is produced by the oxidation of phospholipids in a given tissue [67]. In all treatments, 8-isoprostane levels showed a significant increase in comparison to untreated cells (Figure 6F**).** Exposure of A549 and HepG2 cells to AgNPs resulted in greater levels of malondialdehyde (MDA), 8-epi-PGF2α, 8-hydroxy-2′-deoxyguanosine (8-oxo-dG), heat shock protein A1A (HSPA1A), and heme oxygenase 1 (HO-1), indicating that metal nanoparticles such as AgNPs could potentially cause oxidative damage [68]. At the moment, studies addressing the effects of PdNPs + MLT on oxidative stress markers in A549 cells are lacking. Our results indicate that PdNPs + MLT could potentially induce oxidative stress and eventually increase the level of oxidative stress markers such as ROS, MDA, LHP, NO, protein carbonylation, and 8-isoprostane.

### 3.7. Effect of PdNPs + MLT on Antioxidant Marker Levels

**Figure 7:**
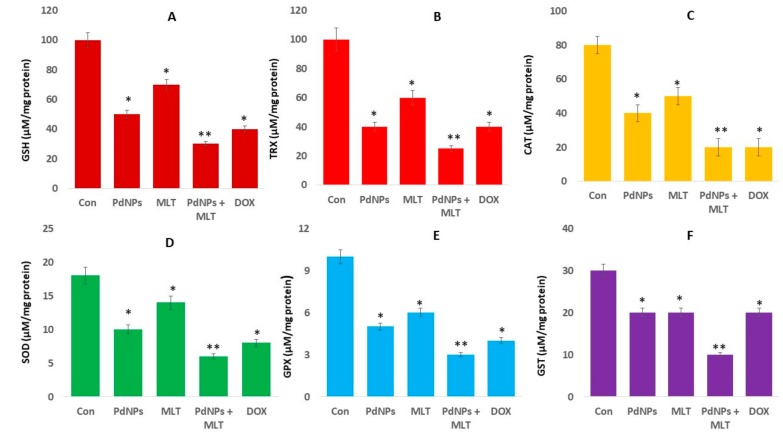
Effect of PdNPs + MLT on antioxidant markers. A549 cells were incubated with PdNPs (2.5 µM), MLT (0.75 mM), PdNPs and MLT (2.5 + 0.75 mM), or DOX (5.0 µM) for 24 h. After incubation, the cells were harvested and washed twice with ice-cold PBS. The cells were collected and disrupted by ultrasonication for 5 min on ice. (**A**). GSH concentration was expressed as micromoles per milligram of protein. (**B**) TRX concentration was expressed as micromoles per milligram of protein. (**C**) CAT was expressed as units per milligram of protein. (**D**) SOD was expressed as units per milligram of protein. (**E**) GPX concentration was expressed as micromoles per milligram of protein. (**F**) GST concentration was expressed as micromoles per milligram of protein. Results are shown as the mean ± standard deviation of three independent experiments. Student’s *t-*test revealed a significant difference between treated and control cells (* *p* < 0.05). * *p* < 0.05 was considered significant; ** *p* < 0.01 was considered highly significant.

PdNPs and MLT show both pro-oxidant and antioxidant properties. However, their effects will depend not only on the concentrations used but also on the type of cells. Intracellular antioxidants such as glutathione (GSH), thioredoxin (TRX), superoxide dismutase (SOD), catalase (CAT), glutathione peroxidase (GPx) and glutathione-S-transferase protect cells against oxidative damage. Changes in the ratio of antioxidants to oxidants have a significant role in the pathogenesis of different cancer types [69]. Enzymatic antioxidants also play a critical role against oxidative stress. Thus, we evaluated the impact of PdNPs and MLT on several antioxidants; treated cells showed reduced levels of all the tested antioxidants, particularly cells treated with the combination of PdNPs and MLT (Figure 7). This result was consistent with the findings of Alarifi et al. [58], who reported that PdNPs significantly reduced the level of GSH in A375 cells.

The same trend was observed in all other antioxidants, including TRX, CAT, SOD, GPx, and GST (Figure 7B–F). Similarly, PtNPs also decreased antioxidant levels in several types of cancer cells, including SH-SY5Y cells [70], human monocytic THP-1 [71], and OS epithelial cells. In mammals, glutathione (GSH) is the dominant low-molecular-weight antioxidant. The ratio between GSH and GSSG maintains the cellular redox potential and redox balance. Under oxidative or nitrosative stress, the GSH/GSSG ratio decreases and can cause neurodegenerative diseases [72]. TRX and GSH are major thiol-dependent antioxidants involved in maintaining cell homeostasis and DNA synthesis and repair [73]. It has been previously reported that phenethyl isothiocyanates may kill malignant cancer cells by disabling the GSH antioxidant system and disrupting redox-sensitive survival pathways [74]. Similarly, PdNPs + MLT decreased the levels of CAT and SOD, which had a significant effect on the rate constant of H_2_O_2_ removal. Additionally, manganese superoxide dismutase (MnSOD), which is involved in ROS homeostasis, exhibits tumor-suppressive and cancer-promoting properties [75]. A reduced level of MnSOD in A431-P and A431-III cells has been shown to promote the metastatic ability of cancer cells [76]. In summary, the mechanism that increases ROS and decreases antioxidants lead to apoptosis in cancer cells.

### 3.8. PdNPs + MLT Caused Mitochondrial Dysfunctions

**Figure 8:**
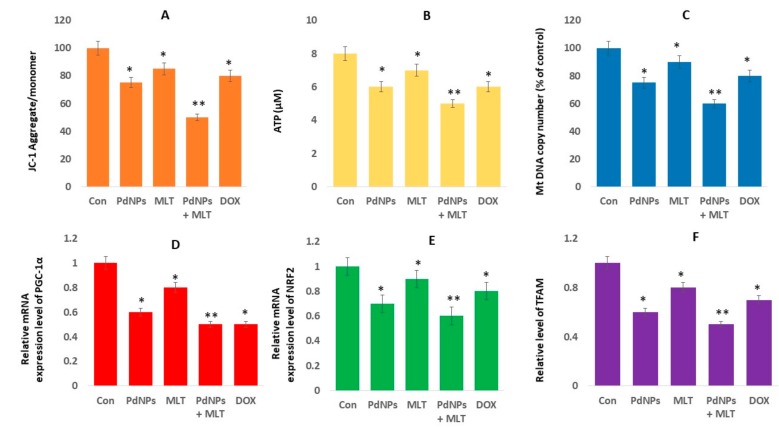
PdNPs + MLT causef mitochondria dysfunctions. Levels of (**A**) MMP and (**B**) intracellular ATP, (**C**) mitochondrial copy number, (**D**) expression level of PGC1α, € expression levels of NRF2, and (**F**) expression levels of TFAM. Results are shown as the mean ± standard deviation of three independent experiments. Student’s *t*-test revealed a significant difference between treated and control cells (* *p* < 0.05). * *p* < 0.05 was considered significant; ** *p* < 0.01 was considered highly significant.

Mitochondria are major sources of free radicals, and are the major sites for melatonin synthesis and metabolism [77]. Several studies have demonstrated that the accumulation of ROS damages mitochondrial function. To determine mitochondrial dysfunction, cells were exposed to PdNPs (2.5 µM), MLT (0.75 mM), PdNPs and MLT (2.5 + 0.75 mM), or DOX (5.0 µM) for 24 h. The loss of mitochondrial membrane potential (MMP) was assessed using a JC-1 assay kit. The MMP was significantly reduced in the treatment combining PdNPs + MLT (up to 20-fold decrease from that in untreated cells) in comparison to both untreated cells and the other treatments (Figure 8A); cells treated with either PdNPs or MLT showed a moderate loss of MMP. Several studies have reported that metal nanoparticles (such as silver, zinc, palladium, and platinum) and graphene oxide silver nanocomposites impair mitochondrial functions by inducing loss of membrane potential in several types of cancer cells, including human alveolar basal epithelial cells [36], human ovarian cancer cells [78], human cervical cancer cells [20], human breast cancer cells [21], and human osteosarcoma cells. Additionally, mitochondrial dysfunction could activate a mitochondrial apoptotic pathway to cause translocation of Bax from the cytosol to the mitochondria [79].

Moreover, ATP production was only moderately affected when cells were treated with PdNPs, MLT, or DOX separately, but decreased significantly in cells treated with PdNPs + MLT (Figure 8B). Furthermore, we examined the ATP generation, which represents the damage to mitochondrial function, under PdNPs or MLT exposure. The results revealed that ATP production was moderately affected compared to under the combined treatment. Combined effects of melatonin and all-trans retinoic acid or somatostatin caused a marked reduction in mitochondrial membrane potential and intracellular ATP production, inducing necrotic cell death [52]. Several metal nanoparticles are known to impair ATP synthesis in mouse embryonic fibroblast cells and human colon cancer cells [80,81], while platinum decreased ATP levels in human alveolar basal epithelial cells [36]. Zhao et al. [82] reported that titanium dioxide nanoparticles induced mitochondrial dynamic imbalance in HT22 cells, eventually causing mitochondrial dysfunctions and decreased level of ATP synthesis. Previous studies have shown that platinum nanoparticles decreased ATP synthesis in human THP-1 [71], prostate cancer cells [70], and osteosarcoma cells. Increased level of ROS reduced the oxidative capacity by altering the oxidative phosphorylation (OXPHOS), and decreased ATP production [83]. Thus, PdNPs and MLT may damage mitochondrial proteins/enzymes, membranes, and DNA, interrupting ATP production and obstructing other essential mitochondrial functions.

We also investigated the combination effect (PdNPs + MLT) on mitochondrial biogenesis, a critical process for metabolic activity. Mitochondrial biogenesis was investigated to evaluate mitochondrial function, a crucial modulator of cellular physiology. Our results indicated that mitochondrial DNA copy number decreased significantly in cells treated with PdNPs + MLT, in comparison to untreated cells and consistent with the results mentioned above. Cells treated with PdNPs or MLT separately also showed a reduced mitochondrial DNA copy number (Figure 8C). It has been previously reported that cells treated with AgNPs + MS-275 exhibited lower mtDNA copy numbers compared to both untreated cells and to cells treated with AgNPs or MS-275 separately. 

Moreover, attention should be given not only to mtDNA copy number but also to its functional status, which depends on mitochondrial biogenesis and dynamics, including fission/fusion and mitophagy. Thus, we measured the main regulators of mitochondrial biogenesis (peroxisome proliferator receptor gamma coactivator-1 alpha - PGC-1α, NRF2, and TFAM). Interestingly, mRNA levels of PGC-1α, NRF2, and TFAM decreased significantly in all treatments (Figure 8D–F), which was incompatible with the mtDNA content. The dysregulation of mitochondrial biogenesis affects mitochondrial volume density and oxidative capacity per mitochondrial volume [84]. The coordination between these processes is controlled by several mutually regulated signaling cascades, one of the most important being the Nrf2/ARE cascade [85]. Studies have reported that NRF1 could bind to specific promoter sites and regulate the expression of TFAM [86], TFB1M, and TFB2M [87], while NRF2 is involved in regulating the expression of other mitochondrial enzymes. MLT regulates the mitochondrial translocation of mitochondrial fission proteins: mitochondrial fission 1 protein (Fis1), dynamin-related protein 1 (Drp1), and the pro-apoptotic protein, Bax [88]. MLT suppresses the translocation of Fis1 and Drp1 to the outer mitochondrial membrane, thus reducing fission. Our results agreed with previous findings reported by Guo et al. [89], which suggested that silica nanoparticles limits the expression of PGC-1α, NRF2, and TFAM in human umbilical vein endothelial cells (HUVECs).

Thus, our results indicate that the combination of PdNPs with MLT potentially limits mitochondrial biogenesis. Although several studies have demonstrated that MLT prevents mitochondria from oxidative damage, at higher concentrations, ROS production takes place [90]. In summary, our findings indicate that both PdNPs and MLT may be involved in mitochondrial biogenesis by regulating genes involved in mitochondrial biogenesis. Disruption of the mtDNA copy number could influence the mitochondrial function and could have a significant impact on the general functions of cells.

### 3.9. PdNPs + MLT Induced Apoptosis and Oxidative DNA Damage

**Figure 9:**
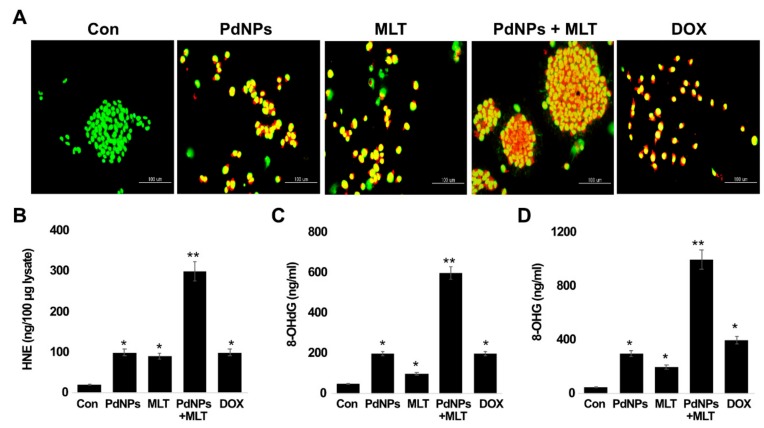
PdNPs + MLT induce apoptosis and oxidative DNA damage. (**A**) Morphological observation of cells under each treatment. Green staining indicated viable cells, whereas yellow-stained cells were early apoptotic and reddish- or orange-stained cells were late apoptotic. Each experiment was performed in triplicate (*n* = 3) and similar morphologic features were observed. Original magnification 200 μm. Levels of (**B**) HNE, (**C**) 8-Oxo-dG, and (**D**) 8-Oxo-G level. Results are shown as the mean ± standard deviation of three independent experiments. Student’s *t-*test revealed a significant difference between treated and untreated cells (* *p* < 0.05) and between the treated and control cells. * *p* < 0.05 was considered significant; ** *p* < 0.01 was considered highly significant.

To confirm that apoptosis was induced by PdNPs and MLT, A549 cells were treated with PdNPs (2.5 µM), MLT (0.75 mM), or PdNPs and MLT (2.5 + 0.75 mM) for 24 h and analyzed for oxidative DNA damage and apoptosis in the presence of acridine orange/ethidium bromide staining (AO/EB staining). The assay specifically investigated the morphology of the nucleus, specifically for signs of apoptosis. The live cells showed normal nuclear chromatin with green fluorescence, but apoptotic cells contained fragmented DNA (intense orange color). We observed a large number of apoptotic cells in all treatments in comparison to untreated cells, particularly in the treatment combining PdNPs with MLT (Figure 9A). Cells stained green were viable, yellow staining represented early apoptotic cells, and reddish or orange staining represented late apoptotic cells. A549 cells treated with PdNPs + MLT showed significant changes in cell morphology, including chromatin condensation, membrane blebbing, and fragmented nuclei, indicating that PdNPs + MLT may induce early and late apoptotic stages. Similar features were also observed in cells treated with PdNPs, MLT, and DOX separately. In addition, the combination treatment exhibited severe damage and additional features of late apoptosis. Cells treated with PdNPs, MLT, PdNPs + MLT, or DOX were more prone to chromatin condensation, nuclear fragmentation, and apoptotic bodies compared to untreated cells. Several studies have reported that metallic nanoparticles can potentially induce apoptosis via DNA chromatin condensation and fragmentation in several types of cancer cells, including human breast cancer cells, ovarian cancer cells, human neuroblastoma cancer cells [91], and human bone OS epithelial cells.

Accumulating evidence suggests that one of the most feasible mechanisms of metallic-nanoparticle-induced apoptosis is the production of ROS, which causes oxidative damage to DNA. Oxidative DNA damage includes oxidized DNA bases and single-stranded or double-strand DNA breaks. The hypothesis that oxidative stress mediates accumulation of 4-HNE was tested using ELISA. Since oxidative stress is a common feature of NPs, we tested the effects of PdNPs + MLT on DNA. The combination treatment (PdNPs + MLT) showed significantly higher accumulation rates of 4-HNE (300 μg/100 μg lysate) compared to cells treated with PdNPs (100 μg/100 μg lysate) or MLT (90 μg/100 μg lysate) separately (Figure 9B). The positive control, DOX-treated cells, showed an accumulation rate of 100 μg/100 μg lysate.

Guanine is the nitrogen base most susceptible to oxidation by ROS, as 8-oxoguanine (8-oxoG) is formed. Thus, we examined levels of 8-OHdG and 8-OHG, important oxidative stress markers. Cells treated with PdNPs + MLT had an accumulation rate of 8-OHdG (600 ng/mL) that was significantly higher compared to cells treated with PdNPs (200 ng/mL) or MLT (100 ng/mL) separately (Figure 9C,D). For example, BALB/c 3T3 fibroblast cells exposed to gold nanoparticles showed DNA damage through an indirect mechanism triggered by oxidative stress [92]. Cells exposed to PdNPs and MLT separately and cells exposed to PdNPs + MLT exhibited severe oxidative damage to both DNA and RNA through the accumulation of 8-OHdG and 8-OHG. Similarly, silver nanoparticles caused severe oxidative damage to DNA and accumulation of 8-OHdG and 8-OHG in embryonic fibroblast cells of mice [80], while TiO_2_NP induced ROS-mediated oxidative stress, the activation of p53, Bax, and caspase- 3, and caused oxidative DNA damage in HEK-293 cells [93]. Likewise, human hepatocyte and embryonic kidney cells exposed to zinc oxide NPs (ZnONPs) showed altered cell morphology, mitochondrial dysfunction, increase of oxidative stress markers, and oxidative DNA damage [94]. HaCaT cells treated with silicon dioxide NPs exhibited increased level of ROS, DNA damage, and apoptosis [95]. Finally, Alarifi et al. [58] reported that PdNPs potentially induce apoptosis and oxidative DNA damage in A375 cells. Thus, PdNPs induce cytotoxicity and genotoxic effect in A549 cells, as other metallic NPs have been found to induce oxidative DNA damage in human ovarian cancer cells [22] as PtNPs have in human monocytic THP-1 cells [71]. The combination of PtNPs and doxorubicin caused significant oxidative DNA damage and accumulation of 8-OHdG and 8-OHG in osteosarcoma cancer cells. Iavicoli et al. [50] reported that PdNPs induced single-strand DNA breaks in Rat-1 and A549 cell lines. PdNPs inhibited cell growth in fibroblasts and lung epithelial cells and induced apoptosis and DNA damage. The treatment of MLT combined with adipose-derived mesenchymal stem cell (ADMSC)-derived exosomes reduced the number of circulating inflammatory cells in rats, and the combined Mel–exosome treatment reduced inflammation, oxidative stress, apoptosis, and fibrosis [96]. Our results clearly support the idea that combining PdNPs with MLT can potentially induce DNA damage.

### 3.10. PdNPs + MLT Increased the Expression Levels of Apoptotic Genes and Caspase 9/3 Activity

**Figure 10:**
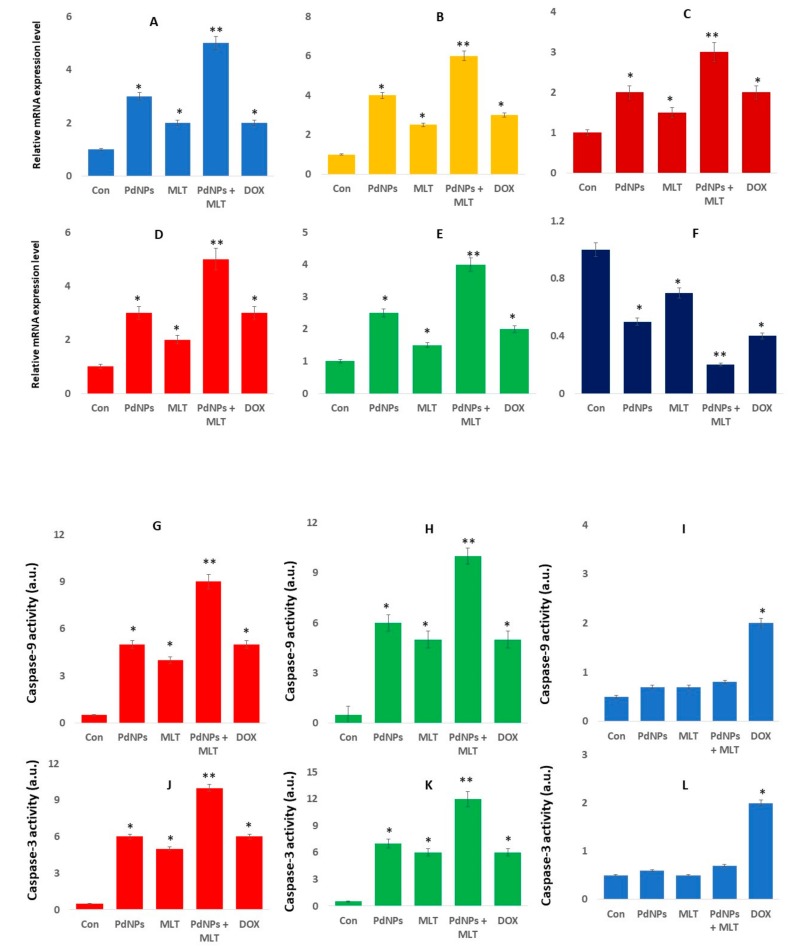
PdNPs + MLT increased the expression of apoptotic genes. The mRNA expression of the apoptotic genes (**A**) *p53* (**B**), *p21* (**C**), *Cyt C*, (**D**) *caspase-3* (**E**) *Bax*, and (**F**) *Bcl-2*. The fold change in the expression was determined relative to GAPDH expression. Caspase 9 activity was determined in A549 human lung cancer cells (**G**), H1229 (**H**), and L132 normal human cells (**I**). Caspase 3 activity was determined in A549 human lung cancer cells (**J**), H1229 (**K**), and L132 normal human cells (**L**). The concentration of the *p*-nitroaniline released from the substrate was calculated from the absorbance values at 405 nm. Results are shown as the mean ± standard deviation of three independent experiments. Student’s *t*-test revealed a significant difference between treated and untreated cells (* *p* < 0.05) and between the treated and control cells. * *p* < 0.05 was considered significant; ** *p* < 0.01 was considered highly significant.

To determine the expression of apoptosis-related genes in the presence of PdNPs and MLT, A549 cells were exposed to PdNPs (2.5 µM), MLT (0.75 mM), or PdNPs and MLT (2.5 + 0.75 mM) for 24 h. Our findings revealed that, in comparison to cells treated with PdNPs and MLT separately, the combined treatment (PdNPs + MLT) increased the expression levels of the following apoptotic genes: *p53, p21, Cyt C*, *caspase-3,* and *Bax* (Figure 10A–E). On the other hand, combining PdNPs with MLT decreased the expression level of the anti-apoptotic gene *Bcl-2* (Figure 10F). Apoptosis induced by the expression of apoptotic genes is essential in chemotherapy, as apoptosis is a highly regulated process of cell death and is activated by several stressors, including cytokines, oxidative stress, and DNA damage [97]. Excessive generation of ROS could explain the increased expression level of apoptotic genes. The *p53* gene is activated in response to a variety of cellular stresses, such as DNA damage, oncogene activation, and nucleotide depletion; moreover, the *p53* gene also activates target genes (i.e., *p21* and *MDM2*), resulting in cell-cycle arrest, senescence, or apoptosis to prevent the proliferation of damaged cells [98,99] Alarifi et al. [58] reported that PdNPs induced cell death in A375 cells by triggering ROS cleavage of caspase-3. PdNPs elicit genotoxic effects by directly interacting with DNA or indirectly via NP-induced oxidative stress and apoptotic responses. Caspase-3 is crucial in apoptosis and is activated by several signals. Additionally, Caspase-3 can cleave a variety of cellular proteins, leading to DNA damage and morphological alterations in cells undergoing apoptosis. It was reported that combining MLT with puromycin increased the activation of caspase-3 and decreased the expression of anti-apoptotic proteins such as bcl-2 and bcl-xL [23]. Likewise, MLT induced pro-apoptotic signaling pathways in human pancreatic carcinoma cells [100], stimulated the expression of *Bad* and *Bax* (pro-apoptotic genes), and enhanced the inhibition of the anti-apoptotic gene *Bcl-2* in human breast cancer cells [101].

Our findings clearly agreed with Kocyigit et al. [51], who found that MLT increased the expression level of P-21, P53, Caspase-3, and Bax protein in human epidermoid carcinoma and normal skin fibroblast cells. However, expression levels were higher in cancer cells than non-cancer cells at higher doses of MLT. DNA damage and fragmentation is one of the vital and irreversible events in apoptosis [102]. Thus, our observations agreed with the results found for TiO_2_NPs in leukemia L1210 cells [103] and PdNPs in human skin malignant melanoma cells [58]. Kocyigit et al. [51] also reported that higher doses of MLT were associated typical apoptotic signs, including chromatin condensation, karyopyknosis, and nuclear fragmentation in human epidermoid carcinoma and normal skin fibroblast cells. Our findings are consistent with recent studies in which MLT has been reported to act as a pro-oxidant and to induce ROS-dependent DNA damage in cancer cell lines [104,105,106]. Our findings were in alignment with reports related to the natural rare earth metal called cerium. Cerium oxide nanoparticles (CNPs) are known to be cell-protective agents like palladium nanoparticles, reducing oxidative stress by scavenging noxious reactive oxygen species. However, CNPs were found to exert strong anticancer activities, maintaining homeostasis of the cancer microenvironment, normalizing stroma–epithelial communication, contrasting angiogenesis, and strengthening the immune response, leading to a reduction of tumor mass in vivo [107]. Studies reported that some instant CNPs act as pro-oxidants, exert selective cytotoxic effects against cancer cells, and sensitize cancer cells to chemotherapy- and radiotherapy-induced apoptosis. CNPs induced apoptosis on melanoma, but not on stroma cells, which was mainly contributed by ROS production and mitochondria dysfunction [108,109]. A study demonstrated that CNPs exhibited toxicity against cancer cells and inhibited metastasis, and that they can manipulate tumor–stroma interactions to reduce tumor progression and invasion [110]. Similar to our findings, CNPs induced apoptosis via activation of caspase-3 and caspase-9 in tumor cells by initiating a mitochondrion-mediated apoptosis signaling pathway [111]. In summary, our results suggest that PdNPs induce cytotoxicity via fragmentation of DNA and activation of apoptotic genes. However, the degree of cytotoxicity depends not only on the physical and chemical properties of NPs, but also on cell type. Based on these findings, we conclude that PdNPs + MLT show significant cytotoxic effects in A549 cells, which could provide initial information to understand the molecular mechanism involved in apoptosis.

To further substantiate our findings that the combination of PdNPs and MLT induced caspase-mediated apoptosis, human lung cancer cells (A549 and H1229 cells) and L132 human normal lung cell were exposed to PdNPs (2.5 µM), MLT (0.75 mM), or PdNPs and MLT (2.5 + 0.75 mM) for 24 h. Our findings revealed that, in comparison to cells treated with PdNPs and MLT separately, the combined treatment (PdNPs + MLT) increased the expression levels and activity of caspase9/3 (Figure 10G–L). Caspase activation was remarkably increased in the presence of PDNPs or MLT, or the combination of both PdNPs and MLT (Figure 10G–L). Mitochondrial alteration plays a major role in apoptosis. The alteration of MMP is responsible for the release of Ca^2+^ and cytochrome *c*, and activation of caspases resulted in cell death [112]. Caspases are primary drivers and are able to cleave key intracellular substrates to promote cell death [113]. Caspases are categorized as initiator (caspase-2, -8, -9, and -10) or effector (caspase-3, -6, and -7) caspases, based on their positions in apoptotic signaling cascades [113]. The results from this study aligned with a previous report that the combination of AgNPs and MS-275 synergistically activated caspase-9 and -3 in A549 cells. The combined effect of AgNPs and MS-275 significantly activated caspase-9 and -3 more than a single treatment [36]. It was confirmed that PdNPs and MLT promoted intrinsic mediated apoptosis in both A549 and H1229 cells, whereas no significant changes was observed in normal human lung cells. Altogether, combination of PdNPs and MLT potentially induces apoptosis through a caspase-mediated pathway.

## 4. Conclusions

Metallic nanoparticles are of great interest given their industrial and biomedical applications. In particular, palladium nanoparticles belong to the platinum group of elements, and are widely employed as an active catalyst material in automotive catalytic converters and within the biomedical industry. Melatonin has a dual role, acting both as a pro- and an antioxidant according to its concentration. Although the introduction of several antitumor agents and treatments has increased survival, undesired side effects are still numerous. Therefore, we investigated the effect of combining PdNPs with MLT on cell viability, cell proliferation, cell morphology, cytotoxicity, oxidative stress, mitochondrial dysfunction, apoptosis, and oxidative DNA damage in A549 human lung cancer cells. Our results indicated that melatonin, and especially the combined treatment (PdNPs + MLT) exerted cytotoxic properties and anticancer effects in A549 cells, providing insights into the potential mechanisms involved in apoptosis. The effects of these compounds on different aspects of lung cancer cells (i.e., cell viability, proliferation, and morphology) suggest their mechanisms of toxicity, such as mitochondrial dysfunction and the activation of pro-apoptotic genes. Combining PdNPs with MLT decreased cell viability and cell proliferation, altered cell morphology, increased ROS production, and increased oxidative stress markers. Conversely, PdNPs + MLT decreased levels of antioxidants that lead to redox imbalance. These findings indicate that both PdNPs and MLT show pro-oxidant cytotoxic effects on A549 human lung cancer cells and, therefore, reduce the viability of cancer cells by activating ROS-dependent cell death, DNA damage, and apoptosis. Furthermore, combining PdNPs with MLT has therapeutic value, as non-toxic concentrations of PdNPs and MLT are more effective, better tolerated and show less adverse effects. Finally, this study suggests that MLT could be used as a supplement in nano-mediated combination therapies used to treat lung cancer. However, confirming the efficiency and safety of such approach in cancer treatments requires further studies.

## Supplementary Materials

The following are available online at https://www.mdpi.com/2076-3921/9/4/357/s1, Table S1: List of primers.
