# Supplementary material for: Melatonin Enhances Palladium-Nanoparticle-Induced Cytotoxicity and Apoptosis in Human Lung Epithelial Adenocarcinoma Cells A549 and H1229
Source: Antioxidants (Basel). 2020 Apr 24;9(4):357. doi: 10.3390/antiox9040357 (PMC7222421; doi:10.3390/antiox9040357)
Supplement: Supplementary file 1 [file antioxidants-09-00357-s001.pdf]

| Gene | List of primers |
|---|---|
| **P53** | F:AGAGACCGTACAGAAGA |
| | R:CTGTAGCATGGGATCCTTT |
| **P21** | F:GTTGCTGTCCGGACTACCG |
| | R:AAAAACAATGCCACCACTCC |
| **Caspase-3** | F:AGGGGTCATTTATGGGACA |
| | R:TACACGGGATCTGTTTCTTTG |
| **Cyt C** | F: GCGTGTCCTTGGACTTAGAG |
| | R: GGCGGCTGTGTAAGAGTATC |
| **Bax** | F:CGAGCTGATCAGAACCATCA |
| | R:GAAAAATGCCTTTCCCCTTC |
| **Bcl-2** | F:TAAGCTGTCACAGAGGGGCT |
| | R:TGAAGAGTTCCTCCACCACC |
| **GAPDH** | F:AGGTCGGTGTGAACGGATTTG |
| | R:TGTAGACCATGTAGTTGAGGTCA |
